# A Multimodal Dataset of Cardiac, Electrodermal, and Environmental Signals

**DOI:** 10.1038/s41597-025-05051-3

**Published:** 2025-05-22

**Authors:** Cezar Anicai, Muhammad Zeeshan Shakir

**Affiliations:** University of the West of Scotland, School of Computing, Engineering and Physical Sciences, Paisley, PA1 2BE United Kingdom

**Keywords:** Scientific data, Occupational health, Technology, Predictive markers, Lifestyle modification

## Abstract

In a rapidly evolving technological landscape across various industries, the emergence of real-time, context-aware solutions for health monitoring holds great promise. The dataset presented here encompasses signals from two domains. Ambient environment signals and physiological responses are captured to provide context for well-being assessment. Cardiac activity and electrodermal activity were selected as health indicators, while indoor ambient conditions were characterized by parameters such as temperature, humidity, light, sound, pressure and air quality as determined by Volatile Organic Compounds (VOCs) and Particulate Matter (PM). Data collection involved 14 participants, with each participant contributing approximately 48 minutes of data. This process resulted in a total of over 600 minutes of data, recorded under varied indoor ambient conditions. This dataset was utilized for classifying ambient environment risks concerning long-term cardiac health and for estimating physiological responses exclusively from ambient environment parameters. The compiled dataset provides opportunities for examining the connections between indoor climates and individuals’ well-being states in diverse environments, thereby enabling additional investigations and applications in the domain of context-aware technology.

## Background & Summary

The well-being and health of individuals can be significantly influenced by environmental conditions^1^. According to the World Health Organization, nearly one in every four deaths can be linked to adverse environmental factors^2^. The conventional approach to acquiring physiological responses, also noted as physiological health indicators, has been tethered to physical connections, providing limited insights into the factors influencing these indicators and posing user comfort and compliance issues^3^. Our study addresses the challenge of monitoring health indicators without the need for direct physical connections to medical devices or wearable sensors, as well as, the challenge to determine with precision how the indoor ambient environment affects one’s health and well-being. In recent studies, physiological responses have served as predictive variables for various health related applications such as assessing cognitive load^4,5^, stress detection^6,7^, thermal comfort estimation^8,9^, emotion classification based on cardiac and electrodermal signals^10,11^ or fatigue detection^12,13^. Despite their application in predicting these outcomes, there is a notable absence of substantial research focused on predicting the physiological indicators themselves. This gap hinders efforts to identify the root causes of health conditions, restraining the transition towards a proactive or preemptive approach to health and well-being.

Datasets have been developed to explore the relationship between emotion classification and physiological signals. One such dataset captures features like skin temperature, electrocardiography, electrodermal activity, impedance signals, blood pressure, cardiac output, and total peripheral resistance^14^. Another study expands on this by collecting electrocardiography, electrodermal activity, respiration, electromyography, and motion data, correlating these physiological parameters with emotions such as calmness, tiredness, tension, excitement, and arousal^15^. Furthermore, physiological parameters including activity, heart rate, respiratory rate, and others have been collected and analyzed to facilitate remote healthcare monitoring for dementia patients^16^. Another dataset provides continuous biometric data such as electrodermal activity, heart rate, and skin temperature, alongside contextual information about stress events^17^. Recent research efforts have been directed towards exploring the relationship between PM10 levels and diverse environmental parameters, encompassing temperature, wind speed, humidity, and aerosol optical depth. These investigations also aim to quantify the potential health benefits associated with mitigating air pollution^18^. Furthermore, analyses of temperature datasets in conjunction with mortality rate data have yielded insights into the correlation between extreme temperatures and mortality rates^19,20^. Additionally, a comprehensive dataset on environmental health determinants, encompassing physical and mental health outcomes and corresponding environmental factors, has been curated and made publicly accessible^21^. Table 1 summarizes works that proposed datasets similar to ours, detailing the recorded ambient environment signals, health signals, proposed applications, and the devices utilized for data collection.

**Table 1:** Available datasets with ambient environment conditions and health indicators data.

Name	Year	Ambient Environment Signals	Health Signals	Application	Devices
En-Gage^32^	2022	motion, indoor temperature, humidity, CO2, noise	electrodermal activity, blood volume pulse, body temperature	cognitive, behavioural, emotion engagement, thermal comfort, arousal, valence	Empatica E4, Netatmo indoor weather station, DigiTech XC0422 outdoor weather station, PHILIO Z-wave
K-EmoPhone^33^	2023	ambient light, ultraviolet light exposure	heart rate, skin temperature, calories burned, electrodermal activity, R-R intervals	affective and cognitive states	Microsoft Band 2, Smartphone
WEEE^34^	2022	pressure, temperature, humidity	O2 consumption, heart rate, respiratory rate, electrodermal activity, skin temperature, heart rate variability	human energy expenditure	VO2 Analyzer, Nokia Earbuds, Empatica E4, Zephyr Bioharness, Wahoo Tickr, Apple Watch, Fitbit Sense, Muse S
Thermal comfort dataset^35^	2023	clothing level, air temperature, relative humidity	skin temperature, metabolic rate, heart rate	thermal comfort prediction	Building-in- -briefcase device^36^, Microsoft Band 2
Health-spa^22^	2024	indoor temperature, humidity, pressure, light, noise, volatile organic compounds, particulate matter	electrodermal activity, electrocardiogram	skin resistance and heart rate estimation, environmental risk classification	MaxEvsys30001, PMS5003, BME680, Grove GSR, VEML 7700, Smartphone

The dataset^22^ we collected encompasses a wide range of ambient environment variables, offering a holistic perspective on monitored conditions. It includes measurements such as ambient light intensity, sound levels, atmospheric moisture, temperature, and barometric pressure. The dataset^22^ also incorporates metrics for indoor air quality, assessing factors like pollutants and ventilation. Airborne particle counts of varying sizes contribute to air quality evaluation. Additionally, physiological variables such as skin resistance, reflecting stress or arousal levels, and heart rate, a vital indicator of cardiovascular health and stress, further enrich the dataset. We termed our dataset^22^ ‘Health-spa’, an acronym representing the signals it includes: Heart, Electrodermal Activity, Light, Temperature, Humidity, Sound, Pressure, and Air Quality. The name also implies its intended use in the context of well-being and wellness, rather than clinical healthcare. A pilot study to determine the feasibility of using indoor ambient conditions to estimate physiological health indicators^23^ was performed on data collected from the principal investigator. Three ML algorithms were trained with 5-fold cross-validation and the results obtained indicated that ambient conditions alone can be utilized to accurately estimate health indicators, as long as the constrains set during data collection are respected.

The value of our dataset^22^ stems from the simultaneous and continuous recording of ambient environment factors and physiological health indicators. One can explore various intelligent analysis objectives, such as regression, classification, or clustering, applied to either joint or individual heart rate and skin resistance data. The raw ECG signal, collected at a sampling frequency of 128Hz, can be processed to extract various features including R-peaks, Q and S waves, the onset and offset points of QRS complex, P and T waves and their onset and offset points^24^. Additionally, more metrics can be derived from ECG signal features, including heart rate variability, fragmented QRS complex, early repolarization, ventricular late potential, index of cardio-electrophysiological balance, heart rate turbulence or T wave alternans^25^. Given the amplitude of the ambient environment factors collected, individual features, subsets of specific features or the entire set of features can be considered as independent or predictor variables.

In summary, our study pioneers a novel methodology for estimating health indicators from indoor ambient data, presenting a paradigm shift towards context-aware monitoring. The implications of this dataset^22^ extend beyond mere observation, by uncovering the intricate links between environmental conditions and physiological responses, we can begin to develop proactive interventions that optimize indoor environments for improved health and well-being. Personalized recommendations for adjusting lighting, temperature or ventilation based on an individual’s real-time physiological responses become attainable. This proactive approach has the potential to mitigate stress, improve cognitive function, and even prevent adverse health outcomes associated with poor indoor environment conditions.

## Methods

This section contains details regarding the procedures employed in acquiring and processing the physiological and ambient environment data collected within our extreme environments chamber. The experiment design, sensor based data acquisition and subsequent computational processing steps are described to ensure the transparency and repeatability of our research. We will provide details on participant demographics, the experimental scenarios employed, the equipment and facilities utilized for data collection, and the data processing methodologies, including data synchronization, concatenation, and cleaning.

The study involved human participants and was therefore reviewed and approved, with application number 18213, by University of the West of Scotland Computing Engineering and Physical Sciences Ethics Committee. All participants were given an information sheet and a consent form outlining the study’s goals, methods, data collection process, and commitment to participant privacy and secure data storage. All participants provided written informed consent for their involvement in the study and the subsequent publication of anonymized data derived from this research.

### Participants

**Fig. 1:**
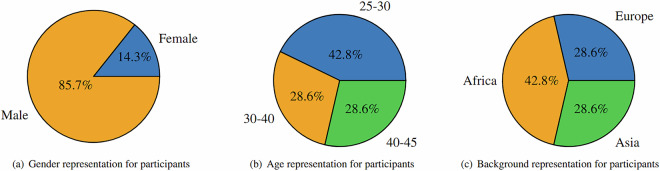
Participant cohort demographic variance.

The study included a cohort of 14 participants, selected to represent diverse demographic characteristics within the constraints of its privacy-preserving design. As shown in Fig. 1, participants spanned multiple age groups and both male and female participants were included. The lack of participants outside the presented age groups and an unbalanced gender representation arose unintentionally due to recruitment biases toward accessible populations. While these demographic gaps may limit analyses of individual differences linked to age or sex, the dataset provides a standardized framework for investigating environmental interactions under controlled yet ecologically valid conditions. Additionally, the cohort comprised individuals from distinct ethnic backgrounds. It is pertinent to note that while these demographic details are pivotal for contextualizing the diversity within our participant pool, it is emphasized that such identifying metadata has not been recorded in the dataset^22^, safeguarding participant anonymity and adhering to ethical research practices.

### Scenarios

Participants were seated at a workstation in the centre of the extreme environments room and were instructed to limit their speech and movements for the duration of the recording, in order to avoid artefacts that would undermine the correlation between ambient environment signals and physiological health indicators. The workstation featured a single board computer connected to an array of sensors for humidity, temperature, pressure, air quality, light, and skin resistance measurements, a mobile device with an application for sound level recording, an embedded board for ECG acquisition, a speaker for noise level adjustment, a laptop and peripheral devices for the single board computer.

Sixteen measurement scenarios were designed, as detailed in Table 2, based on 4 controlled variables. These scenarios represent all unique combinations of values for the chosen controlled variables, calculated using the rule of product. This full factorial design (2^4^ = 16 scenarios) ensures systematic testing of all possible interactions between environmental factors, critical for hypothesis generation and avoiding oversight of potentially relevant correlations. While a fractional factorial design could have improved efficiency, it would sacrifice higher-order interactions–a trade-off incompatible with our exploratory focus on ambient environmental effects. The controlled variables selected were temperature, humidity, light and sound. We did not exert any control on pressure and air quality since the extreme environments chamber was not equipped with the technology necessary, however variations in these signals appeared naturally as a consequence of temperature and humidity adjustments.

**Table 2:** Data collection scenarios.

Artificial Lightening	Noise	Humidity	Temperature	Time
off	45 dBA	40%	25°C	180 seconds
off	45 dBA	40%	35°C	180 seconds
off	45 dBA	65%	25°C	180 seconds
off	45 dBA	65%	35°C	180 seconds
off	85 dBA	40%	25°C	180 seconds
off	85 dBA	40%	35°C	180 seconds
off	85 dBA	65%	25°C	180 seconds
off	85 dBA	65%	35°C	180 seconds
on	45 dBA	40%	25°C	180 seconds
on	45 dBA	40%	35°C	180 seconds
on	45 dBA	65%	25°C	180 seconds
on	45 dBA	65%	35°C	180 seconds
on	85 dBA	40%	25°C	180 seconds
on	85 dBA	40%	35°C	180 seconds
on	85 dBA	65%	25°C	180 seconds
on	85 dBA	65%	35°C	180 seconds

Each scenario lasted for approximately 3 minutes summing to a total 48 minutes of active participation in the experiment required from each participant, excluding the time needed to adjust the environmental conditions within the extreme environments chamber. The time taken to adjust temperature and humidity from one scenario to another was between 15 and 25 minutes depending on the direction of the adjustment, increment or decrement. These gradual transitions replicate real-world environmental dynamics and the response of ventilation systems. In contrast, noise and light were adjusted instantly using a speaker and artificial lighting, simulating abrupt natural changes such as entering a brightly lit room or encountering sudden loud noise.

The sequence of scenario execution was determined by the duration required to stabilize the controlled variables. Given the relatively prolonged adjustment periods for humidity and temperature, we devised four subsets of measurements. Each subset maintained consistent temperature and humidity levels while introducing variations in sound and light parameters. Consequently, participants were tasked with adhering to the experimental protocols continuously for a duration of 12 minutes, constituting one subset of scenarios. Subsequently, they were allotted a relaxation period lasting between 15 to 25 minutes to facilitate adjustments in temperature or humidity levels.

The specific values for the controlled variables were determined following recognized standards and guidelines. For instance, temperature values adhered to the moderate and high recommendations from ISO 7730 standard as identified by^26^, while humidity range aligned with values used in previous studies with comparable temperatures^27^. Noise levels were set in accordance with both UK regulations for workplace noise control^28^ and World Health Organization guidance^29^. Light intensity values were determined during measurements, reflecting ambient lighting conditions in the absence and presence of artificial lighting.

While some residual coupling effects between consecutive scenarios may occur due to the ordering, explicitly documenting the sequence and stabilization intervals allows potential users to consider these dependencies in their analyses, ensuring a robust interpretation that aligns with the study’s real-world-inspired constraints. Our study design aimed to mitigate variability in physiological responses through controlled environmental conditions and a consistent experimental protocol for all participants. This approach helps standardize the context in which physiological signals were recorded and ensures comparability across participants. However, no dedicated effort test or baseline measurement was performed prior to data collection. Depending on the intended application, researchers could perform baseline corrections, normalization techniques, or other post-processing methods to address individual differences. While the controlled conditions and standardized protocol ensure a high degree of consistency in the recorded signals, future studies could benefit from incorporating baseline measurements to further extend the dataset’s applicability.

### Equipment and Facility

**Fig. 2:**
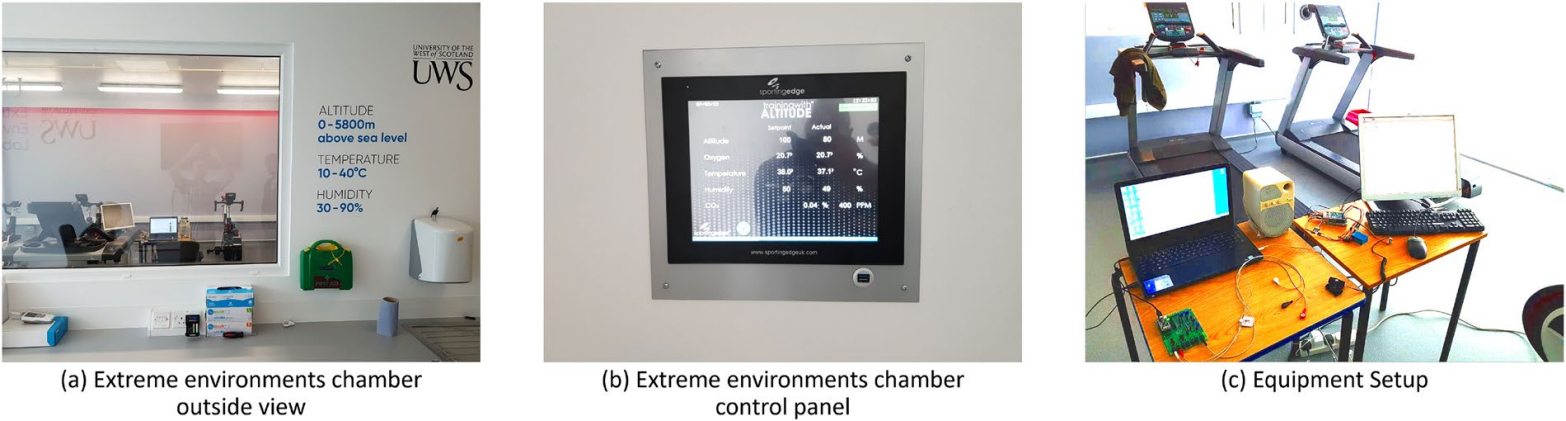
Facilities and equipment used to setup data collection experiments.

The experimental apparatus employed for data collection includes a state-of-the-art extreme environments chamber designed for the precise manipulation of environmental conditions. This chamber facilitates rigorous experimentation by allowing controlled adjustments to temperature, humidity, and oxygen levels, thereby creating extreme environmental scenarios for scientific investigation. The utilization of such a chamber in controlled laboratory settings provides a controlled platform for investigating the nuanced interactions and responses of biological or material entities under extreme conditions. The chamber, it’s control panel and our sensor setup within the chamber are shown in Fig. 2. The environmental chamber can tipically maintain a temperature control accuracy of  ±1 °C and a humidity control accuracy of  ±3%, as specified by the manufacturer, Sporting Edge (UK) Ltd. However, these values represent typical stability intervals rather than absolute limits, meaning minor fluctuations may still occur due to environmental dynamics, sensor placement, and the inherent accuracy limitations of the sensors used for measurement.

**Fig. 3:**
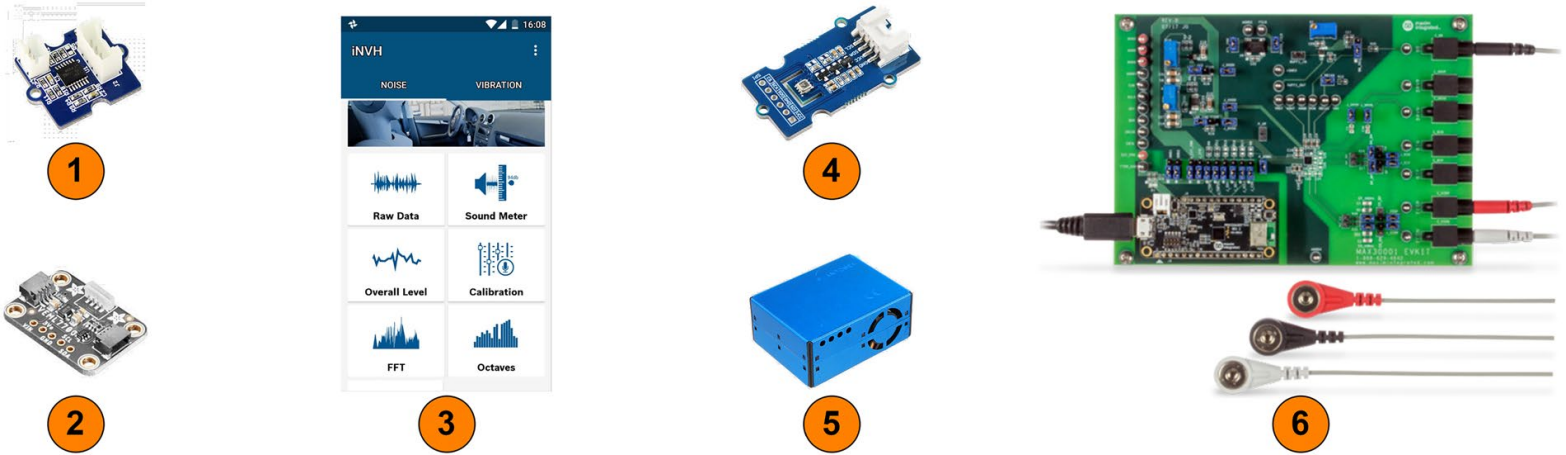
Sensing Devices and Software: (1) Grove GSR Sensor; (2) VEML7700; (3) Bosch iNVH; (4) Bosch BME 680; (5) PLANTOWER PMS5003; (6) Maxim Integrated MAX30001EVSYS.

This project utilizes various sensors to monitor environmental and physiological data, a visual depiction of each component is presented in Fig. 3. The Bosch BME680 sensor acts as a multi-purpose environmental monitor, measuring temperature (ranging from −40 to +85°C), humidity (0 to 100%), pressure (300 to 1100 hPa), and Indoor Air Quality (IAQ) through detection of volatile organic compounds. The accuracy tolerance of the Bosch BME680 sensor for temperature measurements is $\pm 0.5$°C to $\pm 1.0$°C, for humidity the tolerance is $\pm \mathrm{3 \% }$ and $\pm 0.12$ Pa for pressure measurements. The PLANTOWER PMS5003 sensor focuses on airborne particles, providing counts for various sizes particles (greater than 0.3, 0.5, 1, 2.5, 5, and 10 micrometers) within a 0.1-liter air sample. This allows it to estimate concentrations of PM1, PM2.5, and PM10 particles reported in micrograms per cubic meter). Light levels are monitored by the VEML7700 sensor, with a detection range spanning from complete darkness (0 lux) to very bright environments (approximately 120,000 lux). The iNVH android application by Bosch handles noise monitoring, utilizing the built-in microphone of a mobile device (typically MEMS) and offering integrated calibration for various mobile device models.

Moving towards the acquisition of physiological data, the Grove Galvanic Skin Response (GSR) sensor is employed to gauge skin resistance, which serves as an indicator of electrodermal activity, captured by affixing two electrodes onto the index and middle fingers of the left hand. Concurrently, for monitoring cardiac activity, we employed the Maxim Integrated MAX30001 evaluation kit. This kit offers comprehensive data, including raw electrocardiogram (ECG) readings, as well as the calculation of R-R intervals, representing the duration between consecutive heartbeats. The three-lead placement for acquiring cardiac activity data were place on the torso of the participant, following public clinical guidelines^30^.

A single-board computer, Raspberry Pi 3 Model B+, functions as the central processing unit for numerous sensors. It has a 1.4 GHz 64-bit quad-core processor and 1 GB of DDR2 RAM. It provides various connection options, including USB, Bluetooth, Wi-Fi, and a 40-pin General-Purpose Input/Output interface. Streamlining sensor integration is the GrovePi Plus add-on board, which enables communication capabilities via the Inter-Integrated Circuit (I2C) protocol and facilitates the utilization of analog sensors through the incorporation of an analog-to-digital converter (ADC). The method of connection varies depending on the sensor type: I2C for BME680 and VEML7700, analog for Grove GSR, Serial Peripheral Interface (SPI) for PLANTOWER PMS5003, and Universal Serial Bus (USB) for the Maxim Integrated MAX30001 when interfaced with a Windows 10 laptop.

### Data Processing

Since we utilize a Raspberry Pi, a Windows laptop, and a mobile device to integrate sensors, the raw data originates from three distinct sources and it necessitates synchronization based on the timestamp of each sample within the distinct files. Subsequently, all three files are loaded into memory as separate dataframes. We then iterate through each dataframe’s samples to align timestamps and concatenate them feature-wise into a unified dataframe encompassing both predictor and target variables. Outliers were identified and removed on a per-participant basis, based on the Z-score method, which calculates how much a data point deviates from the mean in terms of standard deviations. The mean and standard deviation for each data point were calculated using Eq. 1 and Eq. 2, respectively. Then we subtracted the mean from the value of the data point and divided by the standard deviation to determine the Z-score as described in Eq. (3). We had set a threshold value of +/-3, which considered data points with Z-scores greater than +3 or less than -3 as outliers. Additionally, observations with nan or null values were excluded from the dataset.

1 $$\mu =\frac{{\sum }_{i=1}^{n}{x}_{i}}{n}$$

2 $$\sigma =\sqrt{\frac{{\sum }_{i=1}^{n}{({x}_{i}-\mu )}^{2}}{n-1}}$$

3 $$Z=\frac{x-\mu }{\sigma }$$

The PM sensor collected data on 9 distinct variables including concentration levels for PM1, PM2.5 and PM10, and the count of particles exceeding diameters of 0.3 μm, 0.5 μm, 1 μm, 2.5 μm, 5 μm and 10 μm. However, due to a generally good quality of air at the testing location, concentration levels for PM1, PM2.5 and PM10 and the number of particles with a diameter greater than 2.5 μm, 5 μm and 10 μm were predominantly observed to be zero as shown in Table 3. They were therefore considered redundant and removed from the dataset. However, raw data files are available for independent use or integration. These files can be synchronized and concatenated according to the previously described methods, or they can be employed without following our data processing steps.

**Table 3:** Descriptive statistics for the variables excluded from the dataset.

Variable	Mean	Standard Deviation	Minimum	Median	Maximum
PM1.0 Concentration	0.04	0.31	0.00	0.00	4.00
PM2.5 Concentration	0.05	0.33	0.00	0.00	4.00
PM10 Concentration	0.05	0.33	0.00	0.00	4.00
Nr.particles 2.5 μm	0.16	0.45	0.00	0.00	5.00
Nr.particles 5 μm	0.02	0.14	0.00	0.00	1.00
Nr.particles 10 μm	0.01	0.10	0.00	0.00	1.00

It is pertinent to note that the count of samples in the aggregated dataset, following the preprocessing procedures is 33, 993. The discrepancy between the actual number of data points and the expected count, totaling 6, 327 observations, can be ascribed not only to missing values or outliers, but also to the unavailability of several participants for the entire duration required to complete all scenarios. The expected count derived from the participation of 14 individuals, engaging in 16 scenarios each lasting 3 minutes, with a sampling frequency of 1 Hz, would have amounted to 40, 320 data points.

## Data Records

**Fig. 4:**
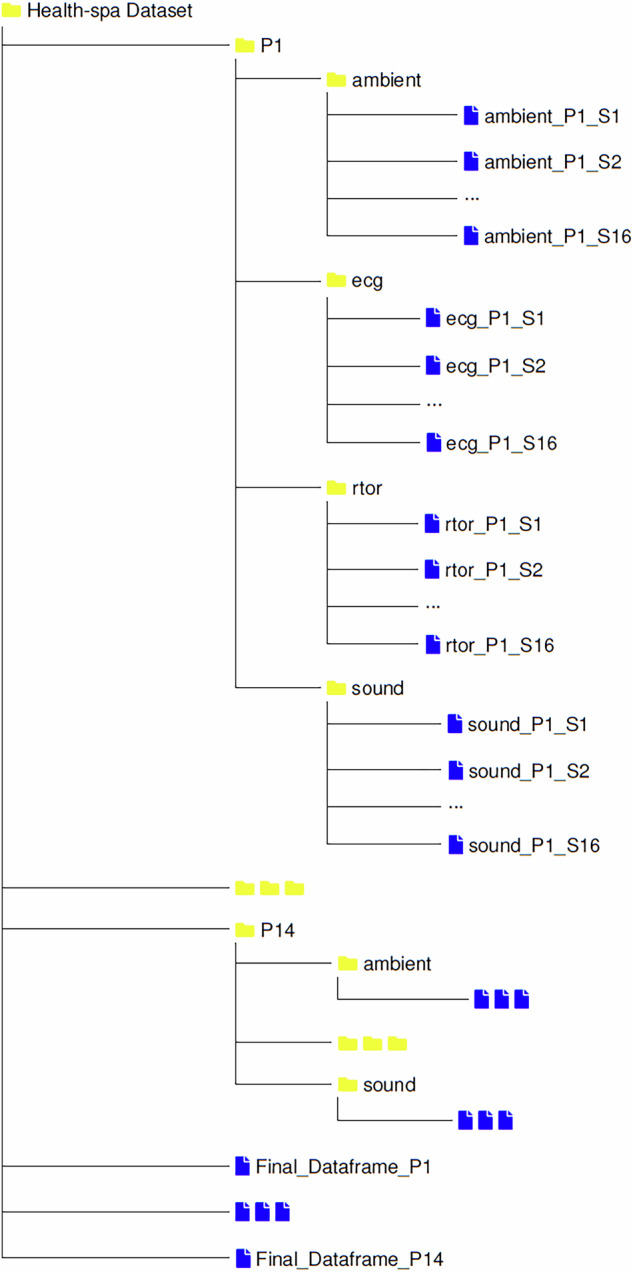
Organization of data files and directories.

The dataset is publicly available and free to download from our figshare repository^22^. The file naming convention is designed to convey the type of data, the participant and the data collection scenario associated with each file. For example sound data for participant number one, for scenario number one is stored in a file named “sound_P1_S1.csv". All of the .csv files containing data samples are explained in detail below and a visual depiction for the organization of files and folders can be seen in Fig. 4.

The file ambient_Pn_Sn.csv comprises sixteen columns, encapsulating all the environmental parameters except sound and also storing measurements for electrodermal activity. The file originates from the Raspberry Pi. The first column in the file corresponds to skin resistance measurements, while the second column denotes light intensity measurements in lux. Subsequent columns three through five provide data on PM1, PM2.5, and PM10 concentrations, respectively. Columns six through eleven represent measurements of the number of dust particles with diameters exceeding 0.3, 0.5, 1, 2.5, 5, and 10 micrometers, respectively. Column twelve is designated for temperature readings, column thirteen for humidity, column fourteen for pressure, and column fifteen for Indoor Air Quality (IAQ). Finally, the last column records the timestamp of each sample.

The file sound_Pn_Sn.csv includes a header containing information about sampling frequency, blocksize, windowing, weighting, averaging, minimum frequency, and maximum frequency. The data within comprises two columns: the first column denotes time, while the second column records sound levels in decibels. The file originates from the mobile device used during data collection.

The next two files pertain to cardiac activity monitoring and were both generated on a Windows laptop. The rtor_Pn_Sn.csv file has two columns, the first one indicates raw R-to-R interval values and the second indicates the corrected time, in seconds, at which the R-peaks occurs. The file ecg_Pn_Sn.csv consists of seven columns. The first column is allocated for time, the second column for ECG_DATA, containing voltage data samples of ECG, and the third column for ECG Data Tags (ETAG). The fourth column is designated for Pace Data Tags (PTAG), while the fifth and sixth columns represent ECG measurements in millivolts before and after filtering, respectively. The last column records the type of filter applied. ETAG values can assume either 000 or 010, denoting that the ECG data for the sample encompasses both valid voltage and time steps within the ECG record. Conversely, values of 001 and 011 indicate transient and invalid voltage information, while the time step remains valid. PTAG values range from 000 to 101 if pace events are detected, while a value of 111 denotes the absence of pace events during the sample recording.

An aggregated dataset file for each participant, Final_Dataframe_Pn.csv, has been compiled for convenience. It comprises eleven columns extracted and concatenated from the raw data files, encompassing all ambient environmental parameters, as well as the two PR variables. Furthermore, all data records have undergone inspection and cleaning processes. Due to variations in sampling rates across the sensing devices, we opted to use the lowest rate when concatenating the files into a single a aggregated file. This approach facilitates seamless integration and simplified analysis across modalities. Importantly, researchers interested in high-resolution analysis can still access the original sensor data with its higher sampling rate.

The following descriptive and visual characteristics of the data have been derived from the observations within all the Final_Dataframe_Pn.csv files. The variables in our dataset^22^ are summarized in Table 4 using descriptive statistics. Each variable includes mean and standard deviation values, determined according to Eqs. (1) and (2), along with the minimum, median, and maximum values. For instance, skin resistance has a mean of 107.9, a standard deviation of 64.62, a minimum value of 20, a median of 100.47, and a maximum of 345.71. Temperature has a mean of 27.64, a standard deviation of 4.54, a minimum value of 20.54, a median of 25.54, and a maximum of 35.06. It is important to note that minor fluctuations in temperature, observed during the experiments, are inherent to the equipment used. The environmental chamber and sensors have accuracy tolerances of  ± 1 °C and  ± 0. 5 °C to  ± 1 °C, respectively. While these fluctuations can yield temperatures below the 25 °C target, they remain within the expected range of variability and do not compromise the validity or reliability of the dataset. The controlled conditions were maintained as rigorously as possible, ensuring consistency across all experimental trials.

**Table 4:** Descriptive statistics for the variables included in the dataset.

Variable	Mean	Standard Deviation	Minimum	Median	Maximum
Skin Resistance	107.9	64.62	20	100.47	345.71
Light	4892.31	4068.71	82	2982	13192
Nr.particles0.3	32.66	59.91	0	15	558
Nr.particles0.5	7.4	9.68	0	4	98
Nr.particles1.0	0.99	1.15	0	1	5
Temperature	27.64	4.54	20.54	25.54	35.06
Humidity	55.17	9.09	39.78	57.68	72.34
Pressure	991.61	16.77	970.4	1000.45	1009.85
IAQ	398336.9	123614.62	311702.74	367736.75	765277.48
Sound	60.73	12.85	45.66	55.61	88.34
Heart Rate	79.37	12.60	25.86	76.81	144.90

**Fig. 5:**
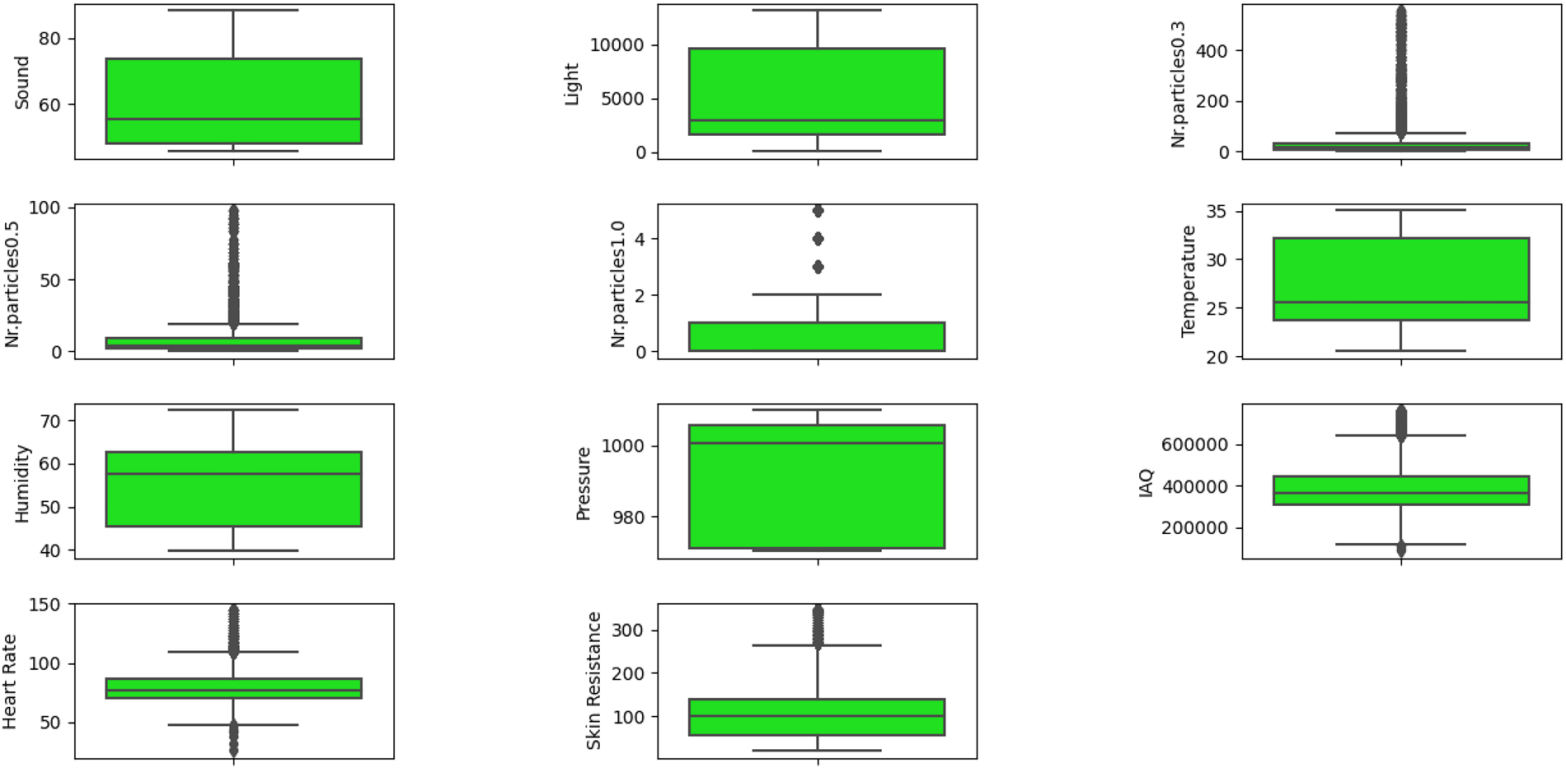
Data points range for all variables across all participants.

The spread and range of values for all features within the dataset^22^ are depicted in Fig. 5. Each box plot’s whiskers extend from the upper and lower quartiles to the furthest data points within 1.5 times the interquartile range (IQR). The box itself represents the range of the central 50% of the data, with the line inside denoting the median value of the feature. The length of the box provides insight into the variability or spread of this central portion of the feature. For instance, heart rate exhibits a range spanning from 70 for the first quartile (Q1) to 90 for the third quartile (Q3). Similarly, sound data ranges between 50 (Q1) and 75 (Q3), while Indoor Air Quality (IAQ) spans from 300,000 (Q1) to 450,000 (Q3). Pressure data ranges from 970 (Q1) to 1,005 (Q3), whereas humidity levels indicate values varying between 45 (Q1) and 60 (Q3). Temperature data ranges from 23 (Q1) to 32 (Q3), and the number of particles data showcases values extending from 0 (Q1) to 36 (Q3). Light data demonstrates values ranging from 1,675 (Q1) to 9,600 (Q3), while skin resistance data reveals values spanning from 60 (Q1) to 145 (Q3).

**Fig. 6:**
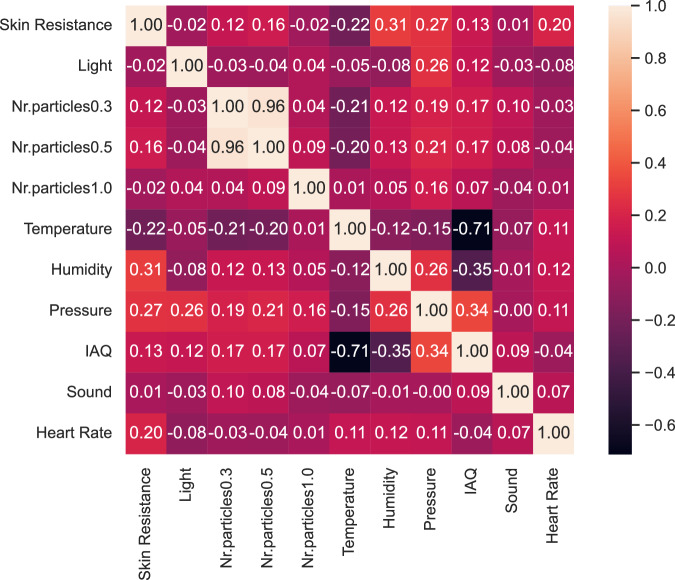
Pearson correlation heatmap for dataset variables.

The relationships between various environmental and physiological parameters within the study are visualized using a Pearson correlation heatmap as shown in Fig. 6. Each cell in the heatmap contains a Pearson correlation coefficient, ranging from -1 to 1, reflecting the strength and direction of the linear association between the corresponding parameters. A correlation coefficient closer to 1 indicates a strong positive correlation, while a value closer to -1 suggests a strong negative correlation. Values near 0 depict a weak or negligible correlation. Upon examination of the correlation matrix, we observe that skin resistance exhibits weak positive correlations with humidity, pressure and heart rate and a weak negative correlation with temperature. Light has a weak positive correlation with pressure, the number of particles with a diameter greater than 0.3 micrometers has a strong positive correlation with the number of particles with a diameter greater than 0.5 micrometers. Temperature exhibits a strong negative correlation with IAQ, humidity has a moderate positive correlation with pressure and a weak negative correlation with IAQ while pressure has a weak positive correlation with IAQ.

## Technical Validation

**Fig. 7:**
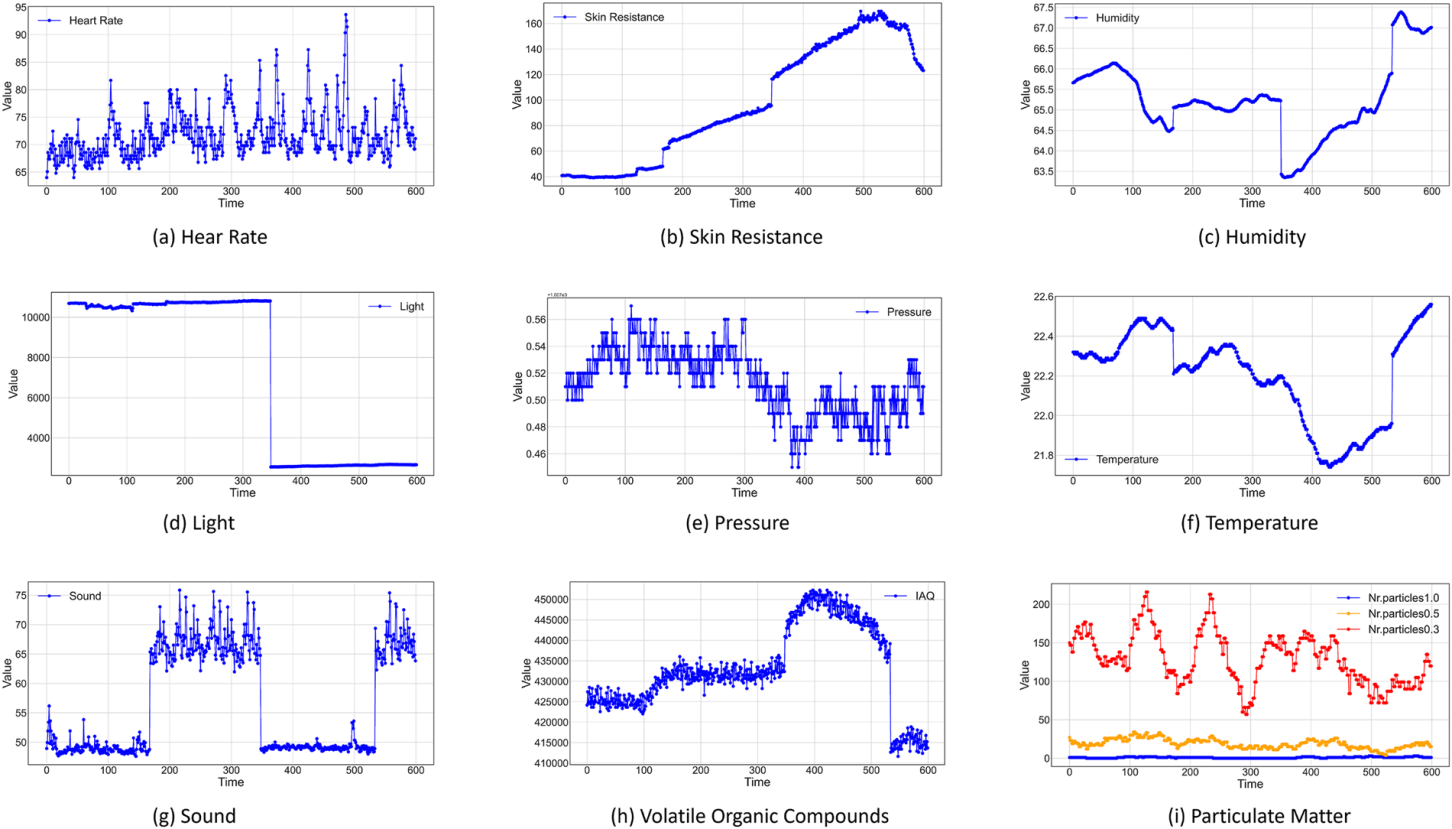
Time series representation of each variable’s values over a period of 600 seconds.

Regarding the technical quality of the dataset^22^, we computed the percentage of missing values for each feature. It was observed that, except for skin resistance, which had approximately 15% missing values, all other variables exhibited only 10 observations with missing values, accounting for less than 0.1%. The line charts presented in Fig. 7 illustrate any existing trend or anomaly in the data for each variable over a period of 600 seconds, encompassing various measurement scenarios. From Fig. 7(d),(g), it is evident that light and sound represent controlled variables, with sound values fluctuating every 180 seconds from around 50dB to around 70dB, and light values changing after approximately 360 seconds from 10,000 lux to under 4,000 lux. Although there is some fluctuation in humidity and temperature Fig. 7(c),(f), the magnitude of change remains under 5%, which can be attributed to the error rate of the extreme environments chamber in maintaining the set parameters. Regarding physiological responses Fig. 7(a),(b), an observed increase over time aligns with fluctuations in ambient signals. As illustrated in Fig. 7(h),(i),(e), air quality, represented by particulate matter and volatile organic compounds, as well as pressure, exhibit variability beyond our control.

In terms of applications, one potential use of the proposed dataset^22^ involves classifying ambient environmental conditions based on their impact on long-term cardiac health. A novel target variable, denoted as “environmental risk” was constructed to encompass three discrete levels: 0, 1, or 2, representing low, medium, and high risk, respectively. This categorization was informed by the associations identified between elevated heart rate exceeding 65 beats per minute (BPM) and the prevalence of cardiovascular disease and mortality, as elucidated in a comprehensive seven-year study involving more than 110, 000 participants^31^.

**Fig. 8:**
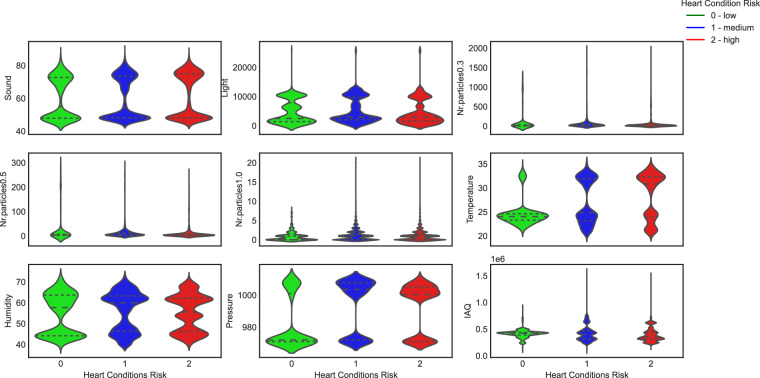
Class distribution among the range of values for each independent variable.

In Fig. 8, the distribution of values for each class, low risk, medium risk, and high risk across all ambient environment variables is shown. Some noticeable patterns include the distribution of classes for temperature values, where temperatures around 25 are more prevalent for the low-risk class. Similarly, for pressure, we observe that medium and high-risk classes are more common around the value of 1000, while the low risk class is more frequently found at 990. In general, the width of the violin at any given point represents the density of data points, with wider sections indicating higher density.

We employed three ML algorithms, Logistic Regression, Random Forest and XGBoost and evaluated them based on precision, recall, F1-Score and accuracy. As it can be seen in Table 5, the linear model was significantly inferior to the tree based models. Random Forest in particular has shown the best performance in almost all classes and metrics, achieving an overall accuracy of 86.5%.

**Table 5:** Low, medium and high risk environment classification results.

Algorithm	Class	Precision	Recall	F1-Score	Overall Accuracy
Logistic Regression	Low risk	0.44	0.02	0.04	0.554
	Medium risk	0.55	0.73	0.62	
	High risk	0.57	0.47	0.52	
Random Forest	Low risk	0.75	**0.68**	**0.72**	**0.865**
	Medium risk	**0.85**	**0.89**	**0.87**	
	High risk	**0.91**	**0.88**	**0.89**	
XGBoost	Low risk	**0.78**	0.64	0.70	0.848
	Medium risk	0.83	**0.89**	0.85	
	High risk	0.89	0.85	0.87	

Another application of the data involves estimating physiological responses, specifically heart rate and skin resistance, solely from ambient environment signals. We utilized the same set of algorithms as in the classification task, with the exception of Logistic Regression, which was replaced by Linear Regression due to the regression nature of the task. Model evaluation was conducted using MAE, RMSE, and MAPE, where lower values indicate superior performance across all evaluation metrics. Analysis of Table 6 reveals that Random Forest emerged as the top performer, achieving an MAE of 2.91 for heart rate and 1.44 for skin resistance. It is noteworthy that the results presented here were derived from models trained on aggregated data from multiple participants. Different outcomes may arise when training individual models for each participant, given the known variability of physiological responses among individuals in the study.

**Table 6:** Heart rate and skin resistance estimation error for models trained on data from all the participants combined.

Algorithm	MAE Heart Rate	RMSE Heart Rate	MAPE Heart Rate	MAE Skin Resistance	RMSE Skin Resistance	MAPE Skin Resistance
Linear Regression	9.60	11.82	0.12	41.68	52.25	0.59
Random Forest	**2.91**	**5.08**	**0.03**	**1.44**	**3.59**	**0.015**
XGBoost	2.94	5.16	0.04	1.68	3.73	0.017

**Fig. 9:**
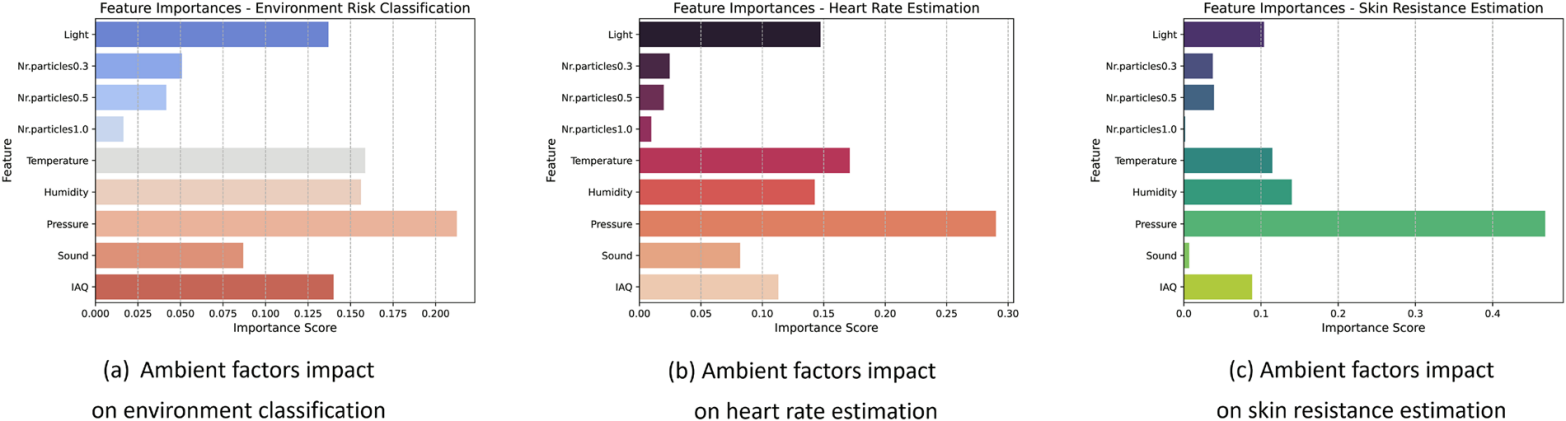
Ambient factors contribution scores for classification and estimation models.

To further support the technical validation of our dataset^22^, we analyzed the contribution of ambient factors to classification and estimation models, as illustrated in Fig. 9. The feature importance scores were computed using the best models for each of the tasks, which in our case are based on the Random Forest algorithm. For the environment risk classification task, Fig. 9a, the most influential features were pressure, temperature, and humidity. In the heart rate estimation model, Fig. 9b, pressure, temperature and light emerged as the top three dominant factors, indicating that variations in these parameters have a measurable impact on physiological responses. Similarly, for the skin resistance estimation model, Fig. 9c, pressure, humidity and temperature were found to be the most influential features. This result highlights the role of atmospheric conditions in modulating skin conductivity, a factor that is well-established in physiological studies. Although some of the top contributing features overlap across tasks, a closer inspection reveals that their importance scores vary significantly between models. This difference in scores demonstrates that ambient factors exert different levels of influence depending on the modeling task, further reinforcing the dataset’s value for enabling tailored analyses across diverse application domains.

## Usage Notes

The dataset^22^ we propose includes measurements for indoor temperature, humidity, pressure, light, sound, air quality, cardiac activity and electrodermal activity. One csv file per participant, synchronized by timestamp and with all the signals concatenated, is available in the repository for direct analysis. The aggregated dataset is suitable for training and evaluating ML or DL models for tasks such as classification, clustering, and regression. We introduced linear and Decision Tree based models for tasks like classifying increased heart rate risk and estimating heart rate and skin resistance. Nevertheless, we encourage peers to delve deeper into these tasks by considering different model approaches.

The raw data files that contain sound measurements, ambient environment and electrodermal activity data and cardiac activity data are also available. Furthermore, additional metrics or features can be extracted from the raw data. For instance, heart rate variability can be readily computed from ECG data, while various ratios between ambient environment features can enhance the dataset’s informational value. We want to emphasize that the raw ECG data was collected at a sampling frequency of 128 Hz, ensuring sufficient temporal precision for heart rate and heart rate variability analysis. Similarly, sound data was recorded at approximately 11 kHz, preserving its detailed temporal structure. The raw data files can be used independently for trend analysis of any of the signals recorded. Investigators have the flexibility to integrate these files using their preferred methods. If a higher sampling rate is needed, interpolation can be applied to increase the resolution of ambient and skin data rather than downsampling the ECG signal.

The 180 seconds recording window in this dataset may not be optimal for all health-related applications, especially those requiring long-term physiological monitoring. However, this duration is well-suited for short-term assessments of stress and physiological responses to environmental conditions.

Given the fact that physiological responses can vary among different individuals and that our dataset^22^ was collected from a number of 14 participants, the influence of sample size on the generalizability of findings should be acknowledged when exploring potential applications. For researchers primarily interested in individual differences, future studies could expand on this work by integrating calibration techniques to account for variability or employing targeted recruitment strategies to fill demographic gaps. Nonetheless, the dataset’s significant range of recorded signals and data points provides a strong foundation for application discovery and hypothesis testing.

Overall the data we provide could contribute to advancements in fields such as environmental science and public health. Employers could use the dataset to optimize indoor workspaces for employee comfort and productivity. Insights from the dataset could inform decisions about lighting, temperature control, acoustic design, and layout to create more comfortable and efficient work environments. The dataset could be leveraged for smart home automation systems to dynamically adjust environmental conditions based on occupants’ preferences and physiological responses. For example, the system could automatically adjust lighting, temperature, and air quality based on user feedback or measured stress levels.

The software packages we used for processing and analysing the data include python programming language, Pycharm and Spyder IDEs and various python libraries. While any programming language and IDE could suffice for data analysis, we especially recommend Spyder for its effectiveness in concatenating raw data files. Spyder offers a notably beneficial Variable Explorer feature, enabling interactive browsing and management of the objects and data structures produced by the code.

## Data Availability

The code used for the technical validation of the dataset^22^ including the classification task and regression tasks is available on the following GitHub repository https://github.com/TheCaesar13/Health-Spa.
